# An Information-Theoretic Perspective on the Quantum Bit Commitment Impossibility Theorem

**DOI:** 10.3390/e20030193

**Published:** 2018-03-13

**Authors:** Marius Nagy, Naya Nagy

**Affiliations:** 1 College of Computer Engineering and Science, Prince Mohammad Bin Fahd University, Al Khobar 31952, Saudi Arabia; 2 School of Computing, Queen’s University, Kingston, ON K7L 2N8, Canada; 3 College of Computer Science and Information Technology, Imam Abdulrahman Bin Faisal University, Dammam 34212, Saudi Arabia

**Keywords:** quantum information theory, bit commitment, protocol, entropy, entanglement

## Abstract

This paper proposes a different approach to pinpoint the causes for which an unconditionally secure quantum bit commitment protocol cannot be realized, beyond the technical details on which the proof of Mayers’ no-go theorem is constructed. We have adopted the tools of quantum entropy analysis to investigate the conditions under which the security properties of quantum bit commitment can be circumvented. Our study has revealed that cheating the binding property requires the quantum system acting as the safe to harbor the same amount of uncertainty with respect to both observers (Alice and Bob) as well as the use of entanglement. Our analysis also suggests that the ability to cheat one of the two fundamental properties of bit commitment by any of the two participants depends on how much information is leaked from one side of the system to the other and how much remains hidden from the other participant.

## 1. Introduction

Bit commitment refers to a cryptographic protocol that can be described informally as follows. In the first phase, Alice decides on a binary value (0 or 1) that she locks in a safe, keeping the key for herself and then hands over the locked safe to Bob. This is referred to as the commit phase of the protocol. Later on, during the decommit phase, Alice reveals her commitment by presenting Bob with the safe key. Bob can now use the key to unlock the safe and learn the value of the bit that Alice has previously committed to.

Any secure implementation of bit commitment must satisfy two crucial properties:

1. Alice should no longer be able to change the value of her commitment, once the safe is in Bob’s hands. This requirement is known as the binding property of bit commitment.
2. Bob should not be able to learn the content of the safe before the decommit phase. This requirement is known as the hiding property of bit commitment.

If Alice and Bob employ quantum means to work out the details of a bit commitment procedure, then the resulting protocol falls into the category of quantum bit commitment (QBC). Unconditionally secure QBC is known to be impossible [1], meaning that no protocol can ever be devised such that both binding and hiding properties are guaranteed, if no restriction is placed on the capabilities of the two participants, Alice and Bob. This result is a direct consequence of the Schmidt decomposition theorem for composite systems [2], but the essence of the impossibility theorem for QBC is not easy to grasp and understand intuitively, beyond the technical details outlined in the papers of Mayers [3] and Lo and Chau [4]. That explains why even after the publication of the “no-go” theorem for QBC, quantum cryptographers were still looking for a protocol that would not fall under its scope. Therefore, in the present investigation, we wish to gain further insight into why hiding and binding are mutually exclusive properties for QBC by adopting the perspective and tools of quantum information theory.

The remainder of this paper is organized as follows. The next section summarizes the most important turns and results that have shaped the history of quantum bit commitment. Section 3 defines a general framework for QBC protocols and arrives at a formulation of the hiding property in terms of the entropy accumulated within the quantum safe. In Section 4, we show that entanglement is a necessary condition for cheating the binding property, using the tools of quantum information theory. A detailed procedure of how Alice is able to cheat the binding property is described in Section 5, with a concrete exemplification for the QBC protocol proposed in the BB84 paper [5]. Section 6 extends the analysis of the two security properties of bit commitment to protocols initiated by Bob. Finally, Section 7 concludes the paper with a summary of our findings.

## 2. Brief History of Quantum Bit Commitment

The feasibility of quantum bit commitment has huge implications for the field of cryptography. Classical cryptography is able to offer solutions (based on bit commitment) to a wide variety of situations classified as discreet decision problems [6]. All these situations share an important characteristic, namely that discretion is vital to achieving agreements. Examples range from negotiating arms treaties to forming business partnerships or organizing mergers.

Classic cryptographic solutions to these applications do exist, but since they involve public-key systems, they are inevitably based on unproven assumptions about the difficulty of factoring large numbers and other related problems. What was expected from quantum cryptography was a totally secure system, guaranteed by the laws of physics, similar to what was already achieved in the case of quantum key distribution [7]. To this end, Claude Crépeau and Joe Kilian have shown how oblivious transfer (or 1-out-of-2 oblivious transfer) can be used as a building block for solving two-party problems requiring discretion [8]. In turn, to provide totally secure quantum oblivious transfer, one would need a secure form of bit commitment. Consequently, much of the research effort in quantum cryptography in the early 1990s was devoted to finding a protocol for quantum bit commitment that is absolutely and provably secure. That result (known as BCJL after the authors’ names) was reported in 1993 [9] and became the foundation for numerous applications in quantum cryptography, pertaining to discreet decision making.

The surprise came in 1995 when Dominic Mayers discovered how Alice could cheat in the BCJL bit commitment protocol by using entanglement [1]. Furthermore, Mayers [3] and, independently, Lo and Chau [4] proved that it would be possible for Alice to cheat in any protocol for quantum bit commitment that guarantees the hiding property. An intuitive explanation is that the description of the quantum safe she hands over to Bob must give nothing away about the committed bit inside. Consequently, regardless of the particular bit commitment scheme employed, the quantum states of the safe containing either 0 or 1 must be very similar (if not identical) since otherwise Bob would be able to discern the difference and gain knowledge about the committed bit prematurely. However, the very fact that the two states are virtually the same gives Alice the possibility to keep her options open and postpone her commitment for later on. Although in their 1996 review paper of quantum cryptography, Brassard and Crépeau [10] argued that for the time being the practical implications of the flaw discovered in the quantum bit commitment protocol are minimal, the weakness definitely affected the entire edifice of quantum cryptography built upon quantum bit commitment.

Perhaps the importance of bit commitment for the general field of cryptography or the intuition that the success of quantum key distribution could be replicated for quantum bit commitment still pushed people to look for a solution. Several protocols were proposed that try to restrict the behavior of the cheater in some way so as to obtain a secure bit commitment scheme [10,11,12]. It turned out that all these protocols were falling under the scope of Mayers’ impossibility result. Building on Mayers’ work, Spekkens and Rudolph [13] proved that the two fundamental properties of bit commitment, binding and hiding, are mutually exclusive. The more a protocol is hiding, the less it is binding and vice-versa. This led to a general belief that the principles of quantum mechanics alone cannot be used to create an unconditionally secure bit commitment protocol. Therefore, recent advances on the topic either exploit realistic physical assumptions like the dishonest party being limited by “noisy storage” for quantum information [14] or combine the power of Einstein’s relativity with quantum theory [15,16,17]. Yet another direction explored by researchers in the field is the class of “cheat-sensitive” quantum bit commitment protocols. Since the hope of designing an unconditionally secure QBC protocol had to be abandoned, researchers focused instead on protocols in which the probability of detecting a dishonest participant is merely required to be non-zero. Properties of such cheat-sensitive protocols are explored in [18,19,20,21,22,23].

Ultimately, secure bit commitment using quantum theory alone remains unattainable. Although we know that entanglement and Schmidt’s decomposition theorem are key ingredients in this impossibility result, these are technical details that fail to provide a deep and intuitive understanding on why, for example, unconditionally secure quantum key distribution is possible, but quantum bit commitment is not. In the following, we try to shed some light into what the conditions for successful hiding and binding properties are and to describe the complex relationship between these properties, with the help of quantum information theory and quantum entropy.

## 3. An Information-Theoretic Formulation of the Hiding Property

In a typical QBC framework, Alice encodes some classical information into a quantum system using one out of two possible encoding bases: $B_{0}$ if she decides to commit to 0 or $B_{1}$ if the commit value is 1. The quantum system represents the safe, while the classical information that Alice keeps secret from Bob plays the role of the key in the description of bit commitment above. In order to prevent Bob from squeezing any information from the quantum safe through some clever measurement, $B_{0}$ and $B_{1}$ must be complementary bases, such as the normal computational base and the Hadamard base. In terms of a practical implementation, these represent the rectilinear and diagonal polarizations of a photon.

However, to characterize the hiding property formally, we also need to define more precisely Alice’s secret key that she encodes in the quantum safe. In general, this key can be any string over a certain alphabet, so without loss of generality, let us assume that the key is a binary string of a certain length *n*. Then the hiding property is an expression of Bob’s total uncertainty over the quantum state of the safe. From his point of view, the safe must be in a complete mixture of all possible bitstrings that Alice might have encoded, regardless of the basis used. This is expressed formally, using density matrices, as follows:

(1) $$\begin{array}{cc} \rho_{0}^{Bob} & =\sum_{k_{i}\in {\left(0|1\right)}^{n}}\frac{1}{2^{n}}|B_{0}\left(k_{i}\right)\rangle \langle B_{0}\left(k_{i}\right)|=\frac{I}{2^{n}}\\ & =\sum_{k_{i}\in {\left(0|1\right)}^{n}}\frac{1}{2^{n}}|B_{1}\left(k_{i}\right)\rangle \langle B_{1}\left(k_{i}\right)|=\rho_{1}^{Bob} \end{array}$$

where $\rho_{0}^{Bob}$ represents Bob’s view of the system when Alice commits to 0, and $\rho_{1}^{Bob}$ represents Bob’s view when Alice commits to 1. Since the density matrices corresponding to Alice’s two commitments are identical, there is not even a theoretical chance for Bob to distinguish between a commitment to 0 and a commitment to 1, no matter what measurement(s) he may try to perform. This uncertainty on Bob’s side can also be quantified using the information theoretic concept of entropy:

(2) $$S\left(B\right)=-\mathrm{tr}\left(\rho_{0}^{Bob}\log\rho_{0}^{Bob}\right)=-\mathrm{tr}\left(\rho_{1}^{Bob}\log\rho_{1}^{Bob}\right)=-\mathrm{tr}\left(\frac{I}{2^{n}}\log\frac{I}{2^{n}}\right)=n$$

In other words, the amount of uncertainty in the quantum state of the safe, from Bob’s perspective, is equal to *n* bits and this is the maximum it can be, given that the key used by Alice is of length *n*. Equation (2) therefore captures the hiding property of bit commitment in the language of quantum information theory.

So far, we have concentrated on Bob’s perspective on the quantum system representing the safe, which is just a part of the whole ensemble Alice–Bob. The other part, which remains in Alice’s possession, contains information about the key chosen by Alice and the bit commitment. The two parts are not independent, as the key together with the encoding basis completely determine the quantum state of the subsystem given to Bob. This means that the conditional entropy S(B|A)=0. For the reader not familiar with the various forms of entropy, we mention that S(B|A) is the amount of information present in system B that does not come from system A, and is defined as:

(3) S(B|A)=S(A,B)−S(A).

In a hypothetical situation, where systems *A* and *B* are completely independent, S(B|A)=S(B). An “entropy Venn diagram” of the whole system depicting the two components and the relationship between them is given below:

The number in each region of the diagram reflects the quantum entropy or amount of uncertainty characterizing the part of the system represented graphically by that region. The mutual information content of *A* and *B*, depicted as the intersection of *A* and *B* in the diagram, measures how much information systems *A* and *B* have in common and is defined as:

(4) S(A:B)=S(A)+S(B)−S(A,B).

Information which is common to both systems is counted twice in the summation S(A)+S(B), while information which is not common is counted just once. Therefore, by subtracting the joint information present in both systems (namely, S(A,B)) from this summation, we obtain just the common or mutual information of *A* and *B*. We note that the whole uncertainty present in subsystem *B* actually comes from subsystem *A* or, equivalently, there are *n* bits of uncertainty shared in the system between the two components (the mutual information S(A:B)=n). On the other hand, subsystem *A* has one more bit of uncertainty compared to subsystem *B*, which is enough to ensure the hiding property, even if Bob is able to dispel the uncertainty characterizing the quantum state of the safe in his view.

It is crucial here to make the observation that the above diagram is constructed solely from Bob’s perspective on the system. Consequently, the fact that S(A|B)=1 cannot be interpreted in the sense that if Bob knows the exact quantum state of the safe, as prepared by Alice, there is still some uncertainty left about the key and/or the bit commitment on Alice’s side. Dispelling the *n* bits of uncertainty describing the quantum state of the safe, simply means that Bob’s knowledge of this state advances from a complete mixture of all $2^{n}$ possible terms to a single, precise, pure state characterizing the quantum safe.

Such a sharp decrease in the entropy of the quantum safe can be triggered, for example, by Bob learning the outcome of a projective measurement applied on all *n* qubits composing the quantum safe, where each possible key $k_{i}$ becomes a projector $P_{i}=|k_{i}\rangle \langle k_{i}|$. However, even after such a measurement is performed, there still remains one full bit of uncertainty about Alice’s choice for the bit commitment and the corresponding key used.

To better understand the point, let us exemplify with the trivial case of a 1-qubit safe and the two encoding bases being the normal computational base and the Hadamard base. In this simple scenario, Alice encodes either 0 or 1 in the basis of her choice and sends the resulting qubit to Bob. Alice keeps a record of the basis chosen (representing her commitment) and the bit encoded (representing the key used). From Bob’s point of view, the whole system is in a mixed state:

(5) $$\rho^{AB}=\frac{1}{4}\left|00\rangle |0\rangle \langle 00|\langle 0\right|+\frac{1}{4}\left|01\rangle |1\rangle \langle 01|\langle 1\right|+\frac{1}{4}|10\rangle \frac{|0\rangle +|1\rangle }{\sqrt{2}}\langle 10|\frac{\langle 0|+\langle 1|}{\sqrt{2}}+\frac{1}{4}|11\rangle \frac{|0\rangle -|1\rangle }{\sqrt{2}}\langle 11|\frac{\langle 0|-\langle 1|}{\sqrt{2}},$$

where the first qubit denotes the encoding basis, the second one represents the encoded key and the third qubit plays the role of the quantum safe passed over to Bob. Thus, the first two qubits come from subsystem *A*, while the last qubit makes up subsystem *B*. The amount of uncertainty present in the system equals two bits, corresponding to the four choices Alice has with respect to the encoding basis and key used:

(6) $$S\left(\rho^{AB}\right)=-\mathrm{tr}\left(\rho^{AB}\log\rho^{AB}\right)=2.$$

In order to see how the quantum safe appears to Bob, we can trace out subsystem *A* from the global state $\rho^{AB}$:

(7) $$\rho^{B}={\mathrm{tr}}_{A}\left(\rho^{AB}\right)=\frac{1}{2}\left|0\rangle \langle 0\right|+\frac{1}{2}\left|1\rangle \langle 1\right|=\frac{I}{2}.$$

This state entails one bit of uncertainty, since it is a mixture of both possible terms |0〉 and |1〉, each with probability 1/2. Naturally, this bit of uncertainty comes entirely from subsystem *A*, as it can easily be checked that the entropy of subsystem *A* equals the entropy of the entire system:

(8) $$\rho^{A}={\mathrm{tr}}_{B}\left(\rho^{AB}\right)=\frac{1}{4}(|00\rangle \langle 00|+\left|01\rangle \langle 01\right|+\left|10\rangle \langle 10\right|+\left|11\rangle \langle 11\right|=\frac{I}{4}.$$

**Figure 1:**
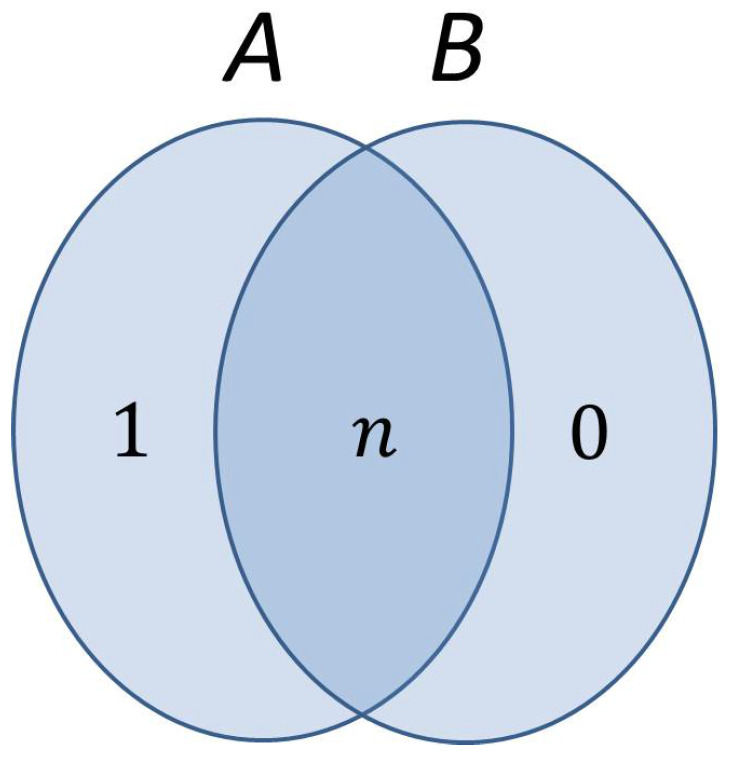
Entropy diagram of the whole system Alice–Bob viewed from Bob’s perspective. All uncertainty present in subsystem *B* (quantum safe) comes from subsystem *A* (Alice’s own quantum register), which has one extra bit of uncertainty.

Dispelling the bit of uncertainty that Bob sees in the quantum safe amounts to Bob being informed or finding out through a measurement which of the two possible pure states the safe actually finds itself in. Regardless of the answer (|0〉 or |1〉), this state can come from both commitments with equal probability, as it can be seen from Equation (5). So the uncertainty on the bit commitment still remains for Bob (S(A|B)=1), thus ensuring the hiding property. On the other hand, if Alice plays by the rules and does prepare a quantum state for the safe based on a specific key and bit commitment, then from her perspective there is not a shred of uncertainty anywhere in the system. All entropies depicted in Figure 1 would be zero in this case.

This is reminiscent of the so-called “relational interpretation” of quantum mechanics (RQM) [24,25], which refutes the idea of an objective or absolute reality (state of a physical system) and proclaims that different observers can give different accounts on the properties of the same physical system. In the case of bit commitment, different accounts arise due to different amounts of information the participants (observers in RQM) have about a particular system, which motivates the use of information theoretic tools in order to get further insight into the problem. It would appear that there is a deep connection between information theory and quantum mechanics which is brought to light in the relational interpretation of the latter.

## 4. Cheating Requires Entanglement

In the previous section, we have clearly stated the condition that needs to be satisfied in order to ensure the hiding property and preclude any possibility of Bob gaining premature knowledge about Alice’s bit commitment. The condition was formulated in terms of both density matrices and using the language of quantum information theory. In this section, we turn our attention to Alice and use the concept of entropy to prove that, if the hiding property is guaranteed, then Alice can cheat and change her commitment in the decommit phase if and only if she is endowed with the ability to generate and manipulate multi-party entangled states.

In a perfectly hiding quantum bit commitment protocol, Alice can cheat in the decommit phase by applying a transformation that will rotate the state of the whole system Alice–Bob from $|\psi_{0}\rangle$ (global quantum state corresponding to a commitment to 0) to $|\psi_{1}\rangle$ (quantum state describing the status of the entire system in the case of a commitment to 1). Since, in the decommit phase, she no longer has access to the safe, which was given to Bob (subsystem *B*), Alice must be able to effect this transformation only by acting on her side (subsystem *A*). This is only possible if subsystem *B* looks the same for both possible commitments, not only for Bob, but from Alice’s point of view as well:

(9) $$\rho_{0}^{Alice}\left(B\right)=\rho_{1}^{Alice}\left(B\right)=\frac{I}{2^{n}}.$$

Then, the transformation $|\psi_{0}\rangle ⟶|\psi_{1}\rangle$ is guaranteed to exist as a direct consequence of the Schmidt decomposition theorem. Condition (9) is not met if Alice prepares the quantum state of the safe using a specific key $k_{i}$:

(10) $$\rho_{0}^{Alice}\left(B\right)=|B_{0}\left(k_{i}\right)\rangle \langle B_{0}\left(k_{i}\right)\left|\neq \right|B_{1}\left(k_{i}\right)\rangle \langle B_{1}\left(k_{i}\right)|=\rho_{1}^{Alice}\left(B\right).$$

Consequently, in such a case, there is no transformation that can rotate $|\psi_{0}\rangle$ into $|\psi_{1}\rangle$ just from Alice’s side. Therefore, maintaining a full uncertainty on the quantum state of the safe is a necessary condition for Alice to cheat:

(11) $$S\left(B\right)=-\mathrm{tr}(\rho^{Alice}\left(B\right)\log\rho^{Alice}\left(B\right))=n.$$

We now show that this condition implies that the entire system Alice–Bob must be in an entangled state.

Regardless of how Alice chooses to prepare the state of the safe (subsystem *B*), she has full knowledge of its state in relation to her own quantum register (subsystem *A*). In other words, since she is the one preparing both subsystems, Alice has complete knowledge of the state of the ensemble Alice–Bob. This means the whole system is in a pure state according to Alice:

(12) S(A,B)=0.

At this point, the Schmidt decomposition theorem can be applied and it follows that both subsystems must have the same eigenvalues. In addition, since quantum entropy is completely determined by the eigenvalues, then it must be the case that the entropy of subsystem *A* is the same as that of subsystem *B*:

(13) S(A)=S(B)=n.

**Figure 2:**
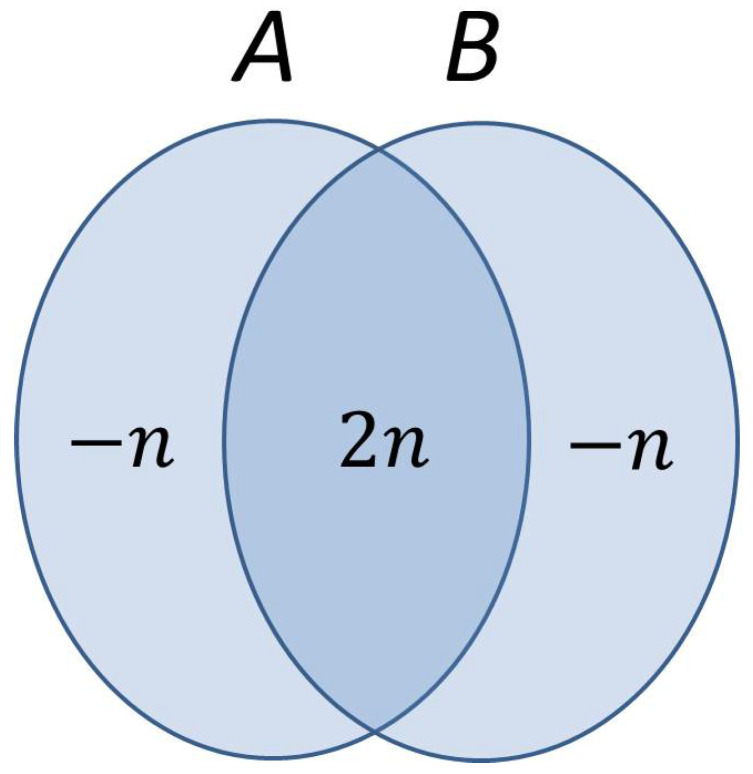
Entropy diagram of the whole system Alice–Bob viewed from Alice’s perspective. The negative conditional entropies indicate that the two components of the system must be entangled.

Based on this last equality and Equation (12), the entropy diagram of the system looks like the one depicted in Figure 2. The mutual information S(A:B)=2n, while both conditional entropies S(A|B)=S(B|A)=−n. A conditional entropy can be negative if and only if the two subsystems are entangled. Equivalently put, a supercorrelation indicated by negative values of conditional entropy is the unmistakable hallmark of entanglement.

In conclusion, we formulate the observation that the binding property can be expressed in the language of quantum information theory as $S_{A}\left(B\right)=0$, forcing Alice to commit to a specific key. A value of *n* for the same entropy guarantees a cheating strategy that completely circumvents the binding requirement of bit commitment. Any intermediate value for $S_{A}\left(B\right)$ (between 0 and *n*) denotes some degree of entanglement between the quantum safe and Alice’s register, which ultimately leads to some probability of cheating from her part and consequently, a partial realization of the binding requirement.

It is interesting to note that, from an information-theoretic perspective, both basic properties of bit commitment are formulated by quantifying the entropy of subsystem *B* (the quantum safe), albeit from two different points of view:

(14) $$S_{B}\left(B\right)=n$$

ensures the hiding requirement by maximizing the entropy of the safe from Bob’s perspective, while

(15) $$S_{A}\left(B\right)=0$$

is the condition that prevents Alice from cheating the binding property by minimizing the entropy of the safe from her point of view.

## 5. Something up Her Sleeve

Having established that entanglement is an essential ingredient in Alice’s cheating strategy, let us now detail how she can take advantage of this important quantum resource in order to avoid commitment and keep her options open until the decommit step.

The initial state of the system prepared by Alice has to take into consideration the two requirements: using entanglement and keeping a full uncertainty on the key used to “lock” the quantum safe. Consequently, Alice prepares an entangled superposition in which each term corresponds to one possible key and the encoding corresponds to a commitment to 0:

(16) $$|\psi_{0}\rangle =\frac{1}{\sqrt{2^{n}}}\sum_{i}|k_{i}\rangle ⊗|B_{0}\left(k_{i}\right)\rangle .$$

The term to the left of the tensor product describes Alice’s own quantum register, while the term on the right characterizes the quantum state of the safe.

This departure from the original protocol in which Alice is supposed to commit to a specific key value is sometimes labeled as the “purified” version of the protocol, due to the fact that the global state of the system is now a pure state. However, this modification which is essential for Alice’s cheating strategy is transparent for Bob. He cannot distinguish between the original and purified version just based on the state of the quantum safe, in both cases the reduced density matrix being the same and equal to $\rho_{0}^{Bob}$ from Equation (1). In a way, this is part of the reason why cheating is always possible in a QBC protocol.

In the decommit phase, if Alice wants to keep her commitment to 0, she just measures her quantum register (in the normal computational basis) and announces to Bob the values obtained as the encoding key. On the other hand, if she wishes to change her commitment to 1, she will have to first apply a transformation on her register that will rotate the overall state of the system to

(17) $$|\psi_{1}\rangle =\frac{1}{\sqrt{2^{n}}}\sum_{i}|k_{i}\rangle ⊗|B_{1}\left(k_{i}\right)\rangle$$

and then apply the measurement. Let us illustrate this point by showing what happens in the case of the QBC protocol proposed by Bennett and Brassard in their seminal paper which launched the field of quantum cryptography [5].

The BB84 QBC protocol adheres to the generic framework outlined at the beginning of Section 3 with the particularizations that base $B_{0}$ represents rectilinear polarization of a photon and $B_{1}$ represents diagonal polarization. For the purpose of a theoretical analysis abstracted away from implementation details, we will use the normal computational basis and the Hadamard basis as $B_{0}$ and $B_{1}$, respectively.

The cheating strategy described in the original BB84 manuscript involves Alice preparing *n* Bell states $\frac{1}{\sqrt{2}}|00\rangle +\frac{1}{\sqrt{2}}|11\rangle$, keeping the first qubit from each entangled state as her own quantum register and sending the second ones to Bob as the quantum safe. Formally, the initial state of the ensemble Alice–Bob is therefore:

(18) $$|\psi_{0}\rangle =(\frac{1}{\sqrt{2}}|0_{A}0_{B}\rangle +\frac{1}{\sqrt{2}}|1_{A}1_{B}{\rangle )}^{⊗n},$$

where the labels *A* and *B* are used to identify the two parts of the system. A closer look to state $|\psi_{0}\rangle$ reveals that it is actually an entangled superposition of all $2^{n}$ possible keys with their encodings in the normal computational basis:

(19) $$|\psi_{0}\rangle =\frac{1}{\sqrt{2^{n}}}\sum_{i}|k_{i}^{A}\rangle ⊗|k_{i}^{B}\rangle ,$$

so Alice is actually preparing the initial state according to the purified version of the protocol as explained above.

Since a Bell state always yields perfectly correlated outcomes when the two qubits are measured in the same basis, regardless of what this basis is, Alice can claim commitment to either of the two possible bit values in the decommit step. This is how cheating is explained in the BB84 paper. Yet again, at a closer inspection, measuring a tensor product of Bell states either in the normal computational basis or in the Hadamard basis conforms exactly to the general cheating procedure described above.

If Alice wishes to claim a commitment to 0, she simply measures her register (subsystem *A*) in the normal computational basis, collapsing the superposition of all possible keys to a specific key $k_{i}$. From Equation (19) it is obvious that when Bob measures his subsystem (the safe) also in the normal basis, he will obtain the same outcome $k_{i}$ due to the entanglement present in state $|\psi_{0}\rangle$.

According to our previously described cheating procedure, if Alice wants to claim a commitment to 1, she has to first rotate the state of the system from $|\psi_{0}\rangle$ to

(20) $$|\psi_{1}\rangle =\frac{1}{\sqrt{2^{n}}}\sum_{i}|k_{i}\rangle ⊗H^{⊗n}|k_{i}\rangle ,$$

by applying a quantum transformation on her register. After that, she can measure the register in the normal computational basis and whatever outcome $k_{i}$ she obtains, it will coincide with the outcome obtained by Bob after a measurement in the Hadamard basis on his side. We claim that the transformation that Alice has to operate on her quantum register before measurement is the Hadamard transform. In other words, the Hadamard measurement invoked in the BB84 paper can be seen as a Hadamard transform followed by a measurement in the normal computational basis.

We can now verify formally that the Hadamard transform does the cheating trick by proving the following equality:

(21) $$\left(H^{⊗n}⊗I^{⊗n}\right)|\psi_{0}\rangle =|\psi_{1}\rangle .$$

One way to prove this is by showing that the dot product between the vectors on the left-hand side and the right-hand side of the above equality is 1:

(22) $$\begin{array}{cc} (|\psi_{1}\rangle ,\left(H^{⊗n}⊗I^{⊗n}\right)|\psi_{0}\rangle ) & =(\sum_{i}\frac{1}{\sqrt{2^{n}}}|k_{i}\rangle ⊗H^{⊗n}|k_{i}\rangle ,\sum_{j}\frac{1}{\sqrt{2^{n}}}H^{⊗n}|k_{j}\rangle ⊗|k_{j}\rangle )\\ & =\sum_{ij}\frac{1}{2^{n}}\langle k_{i}|H^{⊗n}|k_{j}\rangle (H^{⊗n}|k_{i}\rangle {)}^{†}|k_{j}\rangle \\ & =\sum_{ij}(\langle k_{i}|H^{⊗n}|k_{j}{\rangle )}^{2}=\sum_{i=1}^{2^{n}}\sum_{j=1}^{2^{n}}\frac{1}{2^{2n}}=1. \end{array}$$

Therefore, if Alice wants to change her commitment (from 0 to 1), she just has to apply $H^{⊗n}$ on her quantum register and measure it in the normal computational basis or equivalently, measure her register in the Hadamard basis. In general, the transformation that will rotate $|\psi_{0}\rangle$ into $|\psi_{1}\rangle$ depends on the particulars of the respective protocol, but it will always exist as guaranteed by the Schmidt decomposition theorem.

## 6. Extensions to Protocols Initiated by Bob

We have seen that, if the entropy of the quantum safe (as it appears to both participants) is maximal and equal to the length of the key used by Alice, then the hiding property is guaranteed, but Alice can cheat the binding property with certainty, taking advantage of her entanglement with the quantum safe. At the first glance, this may appear as a consequence of the fact that Alice is the one initiating the protocol and having full control over how she prepares the quantum state of the system acting as the safe. In other words, she has all the cards in her hand and this may be perceived as an unfair advantage in realizing an unconditionally secure quantum bit commitment protocol.

Therefore, in this section, we extend the discussion to protocols in which the procedure is initiated by Bob, in the hope to deny Alice any opportunity of acting dishonestly. The main idea of the framework we take into consideration here is that Bob should have a choice in preparing the initial state of the quantum safe, prior to Alice encoding her commitment into the safe through the use of a key. Consequently, the commit phase of the protocol should consist of two steps, the first performed by Bob and the second one by Alice:

1. Bob chooses one of *m* initial states $|\phi_{j}\rangle$, j=1,…,m for the quantum system acting as the safe. He then sends the *n* qubits composing the quantum system over to Alice through a quantum channel.
2. Upon receiving the quantum safe, Alice chooses a key $k_{i}$, i=1,…,p and applies a unitary transformation $U_{0}\left(k_{i}\right)$ or $U_{1}\left(k_{i}\right)$ on the qubits composing the safe, depending on whether she wants to commit to 0 or to 1. Afterwards, she sends the qubits back to Bob through the same communication channel and waits for the decommit phase.

In an effort to keep our model as general as possible, we allow different values for the size of the safe (*n* qubits), number of possible initial states (*m*) and number of different quantum transformations (*p*) that Alice can apply on the system prepared by Bob for a given commitment. The latter also coincides with the number of keys $k_{i}$ that Alice can choose from to encode in the quantum safe. Naturally, some (or all) of these three variables can actually be related to each other, depending on the particular characteristics of a specific protocol.

Since the choice of *j* is intended as a protection against cheating attempts by Alice, states $|\phi_{j}\rangle$, j=1,…,m should be chosen such that they cannot be reliably distinguished. In one possible instance of the protocol, Bob could prepare the initial state of the safe by encoding one of the $2^{n}$ bitstrings of length *n* in one of two complementary bases. This means that Bob is choosing the initial state from a set with $2\times 2^{n}$ elements, thus making *m* equal to $2^{n+1}$.

Similarly, the number of keys *p* Alice is encoding in the quantum safe may also equal $2^{n}$. Each key may again represent a length *n* bitstring such that

(23) $$U_{0}\left(k_{i}\right)=U_{0}\left(b_{n-1}\right)⊗U_{0}\left(b_{n-2}\right)⊗\cdots ⊗U_{0}\left(b_{0}\right),$$

where $U_{0}\left(b_{i}\right)$ is either the identity transformation, if $b_{i}=0$, or a certain single-qubit gate *G*, if $b_{i}=1$. Assuming that Alice’s strategy is to avoid commitment and encode all possible keys in quantum parallel, the entropies of the three subsystems (from the point of view of Alice) are as follows:

(24) $$\begin{array}{c} S(Bob)=\log m=n+1, \end{array}$$

(25) $$\begin{array}{c} S(Alice)=\log p=n, \end{array}$$

(26) $$\begin{array}{c} S(Safe)=n. \end{array}$$

There are n+1 bits of uncertainty on Bob’s side, corresponding to the $2^{n+1}$ choices he has in preparing the initial state of the safe. Similarly, Alice’s quantum register (entangled with the quantum safe) is characterized by *n* bits of uncertainty, reflecting the $2^{n}$ possible keys Alice can encode in the quantum safe. Finally, the quantum safe itself appears to be in a fully mixed state following all possible initial state preparations $|\phi_{j}\rangle$, $j=1,\ldots ,2^{n+1}$ and remains in this state after Alice encodes all possible keys $k_{i}$, $i=1,\ldots ,2^{n}$ in quantum parallel. Consequently, the entropy of the quantum safe is maximal and equal to the number of qubits *n* composing the safe.

The entropy of the global system Alice–Bob-Safe is also n+1, since the state of the entire system (from Alice’s point of view) consists of a different entanglement for each possible initial state $|\phi_{j}\rangle$:

(27) $$\rho_{0}^{global}=\sum_{j=1}^{2^{n+1}}\frac{1}{2^{n+1}}(\sum_{i=1}^{2^{n}}\frac{1}{\sqrt{2^{n}}}|k_{i}\rangle U_{0}\left(k_{i}\right)|\phi_{j}\rangle )(\sum_{i=1}^{2^{n}}\frac{1}{\sqrt{2^{n}}}\langle \phi_{j}|U_{0}^{†}\left(k_{i}\right)\langle k_{i}|).$$

For a fixed initial state prepared by Bob, the ensemble Alice-Safe is described by an entangled state (pure state) with zero entropy. Consequently, the Schmidt decomposition theorem dictates that both subsystems have equal entropy:

(28) $$S\left(Safe|Bob\right)=S\left(Alice|Bob\right)=\frac{n}{2}.$$

**Figure 3:**
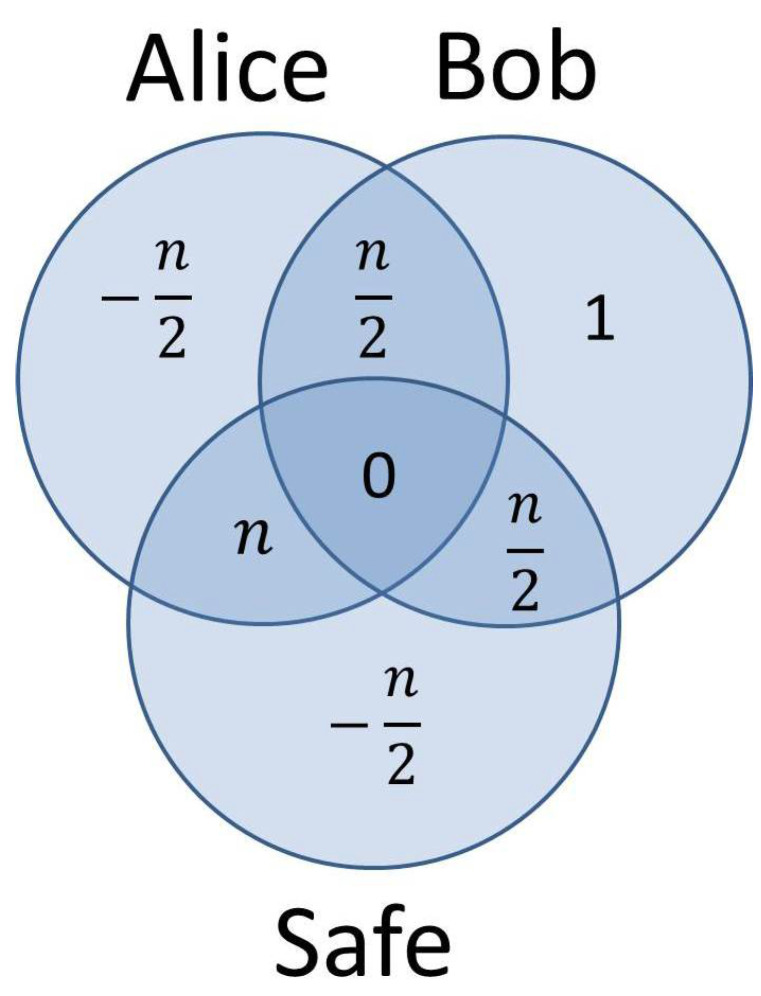
Entropy diagram of the whole system Alice–Bob-Safe viewed from Alice’s perspective. The *n* bits of uncertainty originally encapsulated in the safe by Bob are split between Alice’s quantum register and the safe through entanglement.

The full entropy diagram of the whole system and its components, as it appears from Alice’s perspective, is depicted in Figure 3.

When Bob sends to Alice the *n* qubits representing the quantum safe, the entropy of Bob’s subsystem ((n+1) bits) can be decomposed into *n* bits of uncertainty characterizing the quantum safe handed over to Alice and one bit left for Bob’s own register keeping track of the actual initial state prepared. Subsequently, through the entanglement generated by Alice, the maximal entropy of the quantum safe is spread uniformly among the two entangled parties: Alice’s subsystem (quantum register keeping track of the actual key encoded) and the safe. Consequently, the mutual information between Bob and Alice, respectively, Bob and the safe is

(29) $$S\left(Alice:Bob\right)=S\left(Safe:Bob\right)=\frac{n}{2},$$

while the conditional entropy S(Bob|Alice,Safe) remains 1. We also notice in Figure 3 the negative conditional entropies:

(30) $$S\left(Alice|Bob,Safe\right)=S\left(Safe|Alice,Bob\right)=-\frac{n}{2},$$

revealing the entanglement between Alice’s own quantum register and the quantum safe, entanglement which ultimately allows Alice to steer the state of the system towards a commitment to 1 in the decommit phase, if desired. However, in order for Alice to be able to cheat the binding property, the hiding property must be enforced. In terms of the framework considered in this section, the protocol is hiding if, no matter what its initial state was, the safe looks identical to Bob at the end of the commit phase for both possible commit values:

(31) $$\begin{array}{cc} \rho_{0}^{Bob} & =\sum_{i=1}^{2^{n}}\frac{1}{2^{n}}U_{0}\left(k_{i}\right)|\phi_{j}\rangle \langle \phi_{j}|U_{0}^{†}\left(k_{i}\right)=\\ & =\sum_{i=1}^{2^{n}}\frac{1}{2^{n}}U_{1}\left(k_{i}\right)|\phi_{j}\rangle \langle \phi_{j}|U_{1}^{†}\left(k_{i}\right)=\rho_{1}^{Bob},\;\forall j=1,\ldots ,2^{n+1}. \end{array}$$

In this new scenario in which Bob is the one initiating the protocol, the hiding property is not easy to achieve, since it has to hold for every possible initial state $|\phi_{j}\rangle$. However, one straightforward way to ensure it, is to choose as the single-qubit gate *G* a Hermitian operator (like Hadamard, for example) and then set

(32) $$U_{1}\left(k_{i}\right)=G^{⊗n}U_{0}\left(k_{i}\right),\forall i=1,\ldots ,2^{n}.$$

In this way, when Alice wants to change her commitment to 1 just before the decommit phase, she just needs to flip (apply the negation operator *X* on) all qubits in her quantum register.

For example, consider a three-qubit safe initially in state H|0〉⊗H|1〉⊗H|0〉. Moreover, assume that the key Alice encodes in the state of this quantum safe is 110 by applying the Hadamard gate on the first two qubits of the safe. The state of the quantum safe will consequently change to |0〉⊗|1〉⊗H|0〉. Now, this state is the result of following the procedure corresponding to a commitment to 0. However, we notice that the same state of the quantum safe could have resulted as a consequence of committing to 1, if the encoded key is not 110, but its opposite 001. Furthermore, the same technique of complementing the content of her own quantum register allows Alice to change her commitment regardless of the initial state $|\phi_{j}\rangle$ and/or the encoded key $k_{i}$.

Once again, we can formulate the observation that the entropy of the quantum safe appears maximized (equal to *n*) to both observers. This guarantees the hiding property, but leaves the door wide open for Alice to cheat the binding property of bit commitment, despite the fact that in our new setting Alice is forced to work with a quantum safe whose initial state is not known exactly.

In the setting considered above, Alice is still able to elude the binding requirement at the cost of choosing an operator *G* that is Hermitian. This constraint is a counterpart measure to the advantage that Bob now has through the choice of the initial state for the quantum safe. Certainly, there are other protocol frameworks that can be designed to annihilate Bob’s advantage of trying to prepare an initial quantum state that would allow him to distinguish between the two possible commitments. We describe in the following an alternative protocol that adopts a different approach in enforcing the hiding property for Bob. Subsequently, we analyze the cheating strategies available to Alice and compare the new scenario with the framework we have just investigated, especially from the point of view of entropies and other information-theoretic measures characterizing the various subsystems involved.

In an effort to keep our QBC protocol as simple as possible, our next scenario considers a quantum safe consisting of only one qubit. Bob starts off the protocol by preparing four qubits, one in each of the states |0〉, |1〉, H|0〉 and H|1〉. He sends a random permutation of these four qubits to Alice, who then randomly selects one as the quantum safe. For a commitment to 0, she sends the unaltered qubit back to Bob. A commitment to 1 requires the application of the Hadamard gate before handing Bob the single-qubit safe back.

As in the previous case, we will focus on the purification of the protocol in which Alice acts as if committing to 0, but selects all four qubits received from Bob in quantum parallel. This is done through an entanglement between Alice’s own quantum register (who plays the role of a pointer to the actual qubit selected) and the quantum safe. In order to distinguish among the four possibilities, the quantum register in Alice’s possession must span two qubits. It is easy to check that this protocol is hiding, since, for Bob, the quantum safe appears to be in a fully mixed state, regardless of what particular permutation he is initially sending over to Alice:

(33) $$\begin{array}{cc} \rho_{0}^{Safe} & =\frac{1}{4}\left|0\rangle \langle 0\right|+\frac{1}{4}\left|1\rangle \langle 1\right|+\frac{1}{4}H|0\rangle \langle 0|H+\frac{1}{4}H|1\rangle \langle 1|H=\frac{I}{2}\\ & =\frac{1}{4}H|0\rangle \langle 0|H+\frac{1}{4}H|1\rangle \langle 1|H+\frac{1}{4}\left|0\rangle \langle 0\right|+\frac{1}{4}\left|1\rangle \langle 1\right|=\rho_{1}^{Safe}. \end{array}$$

However, despite the fact that the hiding requirement is realized, Alice can no longer cheat the binding requirement, since in this setting, she needs a different transformation *T* to rotate $|\psi_{0}\rangle$ (global quantum state corresponding to a commitment to 0) to $|\psi_{1}\rangle$ (quantum state describing the status of the entire system in the case of a commitment to 1), for each possible permutation prepared by Bob. For instance, if the four-qubit sequence received from Bob is |0〉⊗|1〉⊗H|0〉⊗H|1〉, then the transformation Alice has to apply on her quantum register is:

(34) $$T_{1}=\left[\begin{array}{cccc} 0 & 0 & 1 & 0\\ 0 & 0 & 0 & 1\\ 1 & 0 & 0 & 0\\ 0 & 1 & 0 & 0 \end{array}\right].$$

However, if Bob prepares and sends the sequence |0〉⊗|1〉⊗H|1〉⊗H|0〉, then it is the following transformation that does the job of rotating $|\psi_{0}\rangle$ to $|\psi_{1}\rangle$ just from Alice’s subsystem:

(35) $$T_{2}=\left[\begin{array}{cccc} 0 & 0 & 0 & 1\\ 0 & 0 & 1 & 0\\ 0 & 1 & 0 & 0\\ 1 & 0 & 0 & 0 \end{array}\right].$$

**Figure 4:**
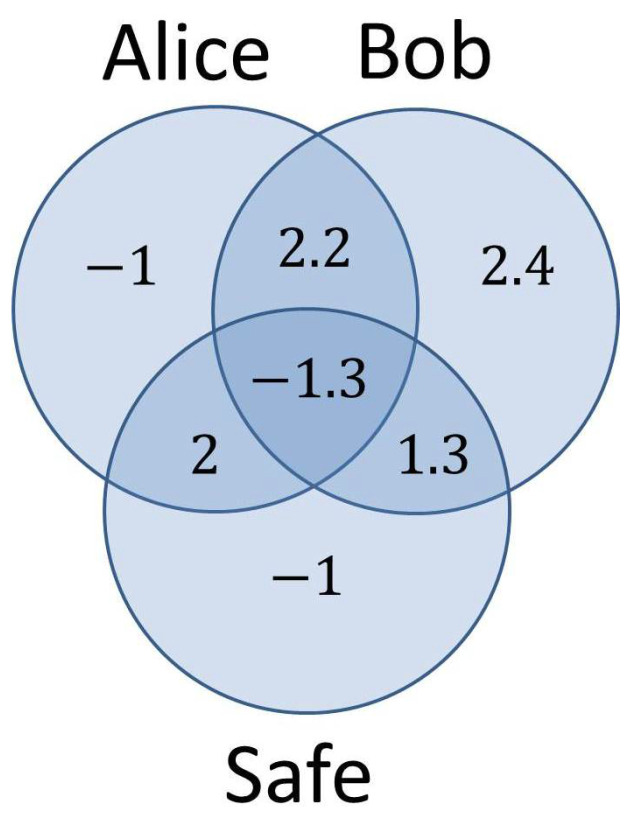
Entropy diagram at the end of the commit phase for an alternative Bob-initiated QBC (quantum bit commitment) protocol, viewed from Alice’s perspective. The amount of uncertainty left in Bob’s subsystem and not shared with the other subsystems prevents Alice from cheating.

Therefore, unlike in the previous instance of QBC, here, there is no unique transformation that can help Alice elude the binding requirement, even though in both protocols she makes use of entanglement in order to apply all her options in quantum parallel. Let us now take a look at the entropy diagram for our current protocol, depicted in Figure 4, and see how this change is reflected in the entropies of the three subsystems involved.

The degree of uncertainty in Bob’s subsystem is log24 (approximately 4.6), corresponding to the 24 degrees of freedom (number of permutations) Bob has in preparing the initial sequence given to Alice. The same value of the entropy also characterizes the entire system, because Alice sees the global state of the system as a mixed state having a different entanglement for each of the 24 possible permutations of the four qubits prepared by Bob.

Assuming that Alice knows exactly the quantum state of each qubit received, the ensemble Alice-Safe is characterized by the exact entangled state corresponding to that particular permutation. This means that the conditional entropy S(Alice,Safe|Bob)=0 and following Schmidt’s decomposition theorem, each side would entail an uncertainty of one bit. As always, the negative conditional entropies S(Alice|Bob,Safe)=S(Safe|Alice,Bob)=−1 witness the use of entanglement by Alice.

Where the entropy diagrams for the two protocols analyzed in this section differ from one another, is the amount of information leaked from Bob towards the other subsystems. In the first case, from the n+1 bits of uncertainty characterizing Bob’s subsystem, almost all of it (*n* bits) gets evenly distributed among Alice and the Safe: $S\left(Alice:Bob\right)=S\left(Safe:Bob\right)=\frac{n}{2}$. Only one bit of information remains unshared with Alice and the Safe: S(Bob|Alice,Safe)=1. In the second protocol, on the other hand, out of the 4.6 bits of information contained in Bob’s subsystem, 2.4 (that is, more than 50%) remains in Bob’s hands only, and does not leak towards the other two subsystems. This is due to the fact that the size of the quantum safe (one qubit) is much smaller than the size of the vector space spanned by Bob’s subsystem (five qubits, in order to accommodate 24 possible initial states). To make a comparison again, there is no mutual information between the safe and Bob’s subsystem in the second scenario, while in the first the two subsystems share $\frac{n}{2}$ bits of information.

The conditional entropy S(Bob|Alice,Safe) measures the amount of uncertainty left in Bob’s subsystem once we acquire all the information in the Alice-Safe ensemble.The magnitude of this entropy seems to be the key in preventing Alice from cheating the binding requirement. Without the knowledge of the particular permutation prepared by Bob (most of which remains in his hands), Alice does not have enough information to rotate the state of the global system towards a commitment to 1.

Does this mean that the particular protocol we have just investigated satisfies both crucial security requirements of bit commitment: it is at the same time binding and hiding? The answer is no, as this would also constitute a counterexample to Mayers’ impossibility theorem on realizing an unconditionally secure quantum bit commitment protocol [1]. The catch is that Bob can also enlist the help of entanglement to his own advantage. He too could not commit to a particular initial state $|\phi_{j}\rangle$ and instead prepare the quantum safe in a superposition of all possible initial states, each entangled with a corresponding label (or pointer) in his own ancilla qubits. This ensemble Bob-Safe would then be in a pure state:

(36) $$|\psi_{init}^{Bob-Safe}\rangle =\sum_{j=1}^{24}\frac{1}{\sqrt{24}}P_{j}(|0\rangle ⊗|1\rangle ⊗H|0\rangle ⊗H|1\rangle )⊗|l_{j}\rangle ,$$

where each $P_{j}$ represents one of the 24 possible permutation operators acting on four qubits. After receiving these four qubits composing the safe from Bob, Alice selects all of them in superposition, effectively creating a three-party entanglement among the three subsystems:

(37) $$|\psi_{0}^{Alice-Safe-Bob}\rangle =\frac{1}{4\sqrt{6}}\sum_{j=1}^{24}\sum_{i=1}^{4}|k_{i}\rangle ⊗U_{0}\left(k_{i}\right)P_{j}(|0\rangle ⊗|1\rangle ⊗H|0\rangle ⊗H|1\rangle )⊗|l_{j}\rangle .$$

In the equation above, $U_{0}\left(k_{i}\right)$, for i=0,1,2,3 is to be interpreted as the selection operator acting on the four-qubit sequence received from Bob whose effect is to select the qubit with index *i*. It is not difficult to verify that by attaching a label $l_{j}$ to each possible permutation $P_{j}$, the hiding requirement is no longer satisfied, as the reduced density matrix obtained from $|\psi_{0}^{Alice-Safe-Bob}\rangle \langle \psi_{0}^{Alice-Safe-Bob}|$ by tracing out the two qubits from Alice’s own quantum register is different from the similar reduced density matrix obtained from $|\psi_{1}^{Alice-Safe-Bob}\rangle \langle \psi_{1}^{Alice-Safe-Bob}|$. Consequently, Bob would be able to distinguish (with a certain probability p>1/2) between a commitment to zero and a commitment to one, which ultimately translates into a non-zero probability of detecting a dishonest Alice.

## 7. Conclusions

The effort that many quantum cryptographers put into developing an unconditionally secure QBC protocol is motivated by (at least) two important factors: the intuition that the success of quantum key distribution can be replicated for bit commitment as well, and perhaps more importantly, the key importance of QBC as a building block in constructing more complex cryptographic applications. These two factors may, at least in part, explain why people kept trying to devise new ways of achieving a protocol with the desired security properties, even after this was proved to be impossible. Another factor may be the relative difficulty in grasping the essence of this impossibility result, especially since other quantum realizations of cryptographic protocols proved superior to their classical counterparts.

In this paper, we adopted a novel approach in trying to get new insight into the problem. With the help of the tools provided by quantum information theory, we have investigated the conditions under which the two fundamental properties of bit commitment, the hiding property and the binding property cannot be reconciled. Our investigation has revealed that both properties can be expressed in terms of the entropy of the quantum system playing the role of the safe into which Alice is supposed to lock her commitment.

The hiding property dictates that the quantum safe must appear to Bob as harboring a certain level of uncertainty. The entropy of the quantum safe is usually maximized (especially when Alice is the one preparing the initial state of the safe) in order to hide the commit value behind the fully mixed state of the quantum system acting as the safe. When dealing with a dishonest Alice, the quantum safe actually achieves maximum entropy with respect to both observers, as Alice needs to keep all her options open. We have also proved that cheating requires enlisting the help of entanglement, a necessary condition without which any cheating strategy is impossible, whether we are talking about breaking the binding requirement by Alice or the hiding requirement by Bob.

Naturally, the ability to circumvent one or the other of the security requirements of bit commitment ultimately depends on the particular details and structure of the protocol at hand. However, our investigation, performed with the specific tools of quantum information theory, seems to point to two important factors influencing the ability to adopt a successful cheating strategy. On one hand, it is easier for any of the two participants to the protocol to mount an effective cheating strategy, if they are the ones initiating the protocol and preparing the initial state of the safe. This gives them the opportunity to avoid committing to a particular initial state and prepare a superposition of all possible initial states, each entangled with a pointer (or label) in their own ancilla qubits. On the other hand, devising a successful cheating scheme or successfully annihilate a cheating attempt by the adversary, also depends on how much information they manage to keep hidden inside their own subsystem and not share it (through the quantum safe) with the other participant.

## References

[1] Mayers D. Unconditionally secure quantum bit commitment is impossible. Proceedings of the Fourth Workshop on Physics and Computation—PhysComp ’96.

[2] Nielsen M.A., Chuang I.L. (2000). Quantum Computation and Quantum Information.

[3] Mayers D. (1997). Unconditionally secure quantum bit commitment is impossible. Phys. Rev. Lett..

[4] Lo H.K., Chau H. (1997). Is quantum bit commitment really possible?. Phys. Rev. Lett..

[5] Bennett C.H., Brassard G. Quantum cryptography: Public key distribution and coin tossing. Proceedings of the IEEE International Conference on Computers, Systems and Signal Processing.

[6] Brown J. (2001). The Quest for the Quantum Computer.

[7] Biham E., Boyer M., Boykin P.O., Mor T., Roychowdhury V. (2006). A proof of the security of quantum key distribution. J. Cryptol..

[8] Crépeau C., Kilian J. Achieving oblivious transfer using weakened security assumptions. Proceedings of the 29th Annual IEEE Symposium on Foundations of Computer Science.

[9] Brassard G., Crépeau C., Jozsa R., Langlois D. A quantum bit commitment scheme provably unbreakable by both parties. Proceedings of the 34th Annual IEEE Symposium on Foundations of Computer Science.

[10] Brassard G., Crépeau C. (1996). 25 years of quantum cryptography. SIGACT News.

[11] Crépeau C. What is going on with quantum bit commitment?. Proceedings of the Pragocrypt ’96: 1st International Conference on the Theory and Applications of Cryptology.

[12] Kent A. (1997). Permanently secure quantum bit commitment protocol from a temporary computation bound. arXiv.

[13] Spekkens R.W., Rudolph T. (2001). Degrees of concealment and bindingness in quantum bit commitment protocols. Phys. Rev. A.

[14] Ng N.H.Y., Joshi S.K., Ming C.C., Kurtsiefer C., Wehner S. (2012). Experimental implementation of bit commitment in the noisy-storage model. Nat. Commun..

[15] Kent A. (2011). Unconditionally secure bit commitment with flying qudits. New J. Phys..

[16] Lunghi T., Kaniewski J., Bussières F., Houlmann R., Tomamichel M., Kent A., Gisin N., Wehner S., Zbinden H. (2013). Experimental bit commitment based on quantum communication and special relativity. Phys. Rev. Lett..

[17] Adlam E., Kent A. (2015). Device-independent relativistic quantum bit commitment. Phys. Rev. A.

[18] Hardy L., Kent A. (2004). Cheat sensitive quantum bit commitment. Phys. Rev. Lett..

[19] Buhrman H., Christandl M., Hayden P., Lo H.K., Wehner S. (2008). Possibility, impossibility, and cheat sensitivity of quantum-bit string commitment. Phys. Rev. A.

[20] Shimizu K., Fukasaka H., Tamaki K., Imoto N. (2011). Cheat-sensitive commitment of a classical bit coded in a block of *m* × *n* round-trip qubits. Phys. Rev. A.

[21] He G.P. (2014). Comment on “Cheat-sensitive commitment of a classical bit coded in a block of *m* × *n* round-trip qubits”. Phys. Rev. A.

[22] Li Y.B., Wen Q.Y., Li Z.C., Qin S.J., Yang Y.T. (2014). Cheat sensitive quantum bit commitment via pre- and post-selected quantum states. Quantum Inf. Process..

[23] Li Y.B., Xu S.W., Huang W., Wan Z.J. (2015). Quantum bit commitment with cheat sensitive binding and approximate sealing. J. Phys. A Math. Theor..

[24] Rovelli C. (1996). Relational quantum mechanics. Int. J. Theor. Phys..

[25] Grinbaum A. (2004). The Significance of Information in Quantum Theory. Ph.D. Thesis.

